# ALK7 expression is specific for adipose tissue, reduced in obesity and correlates to factors implicated in metabolic disease

**DOI:** 10.1016/j.bbrc.2009.03.014

**Published:** 2009-05-01

**Authors:** Lena M.S. Carlsson, Peter Jacobson, Andrew Walley, Philippe Froguel, Lars Sjöström, Per-Arne Svensson, Kajsa Sjöholm

**Affiliations:** a Department of Molecular and Clinical Medicine and Center for Cardiovascular and Metabolic Research, The Sahlgrenska Academy, Vita Stråket 15, 41345 Göteborg, Sweden; b Section of Genomic Medicine, Hammersmith Hospital, Imperial College London, United Kingdom; c CNRS 8090-Institute of Biology, Pasteur Institute, Lille, France

**Keywords:** Adipose tissue, Human, Obesity, Activin receptor-like kinase, Expression, Microarray

## Abstract

Human adipose tissue is a major site of expression of inhibin beta B (INHBB) which homodimerizes to form the novel adipokine activin B. Our aim was to determine if molecules needed for a local action of activin B are expressed in adipose tissue.

Microarray analysis showed that adipose tissue expressed activin type I and II receptors and that the expression of activin receptor-like kinase 7 (ALK7) was adipose tissue specific. In obesity discordant siblings from the SOS Sib Pair study, adipose tissue ALK7 expression was higher in lean (*n* = 90) compared to obese (*n* = 90) subjects (*p* = 4 × 10^−31^). Adipose tissue ALK7 expression correlated with several measures of body fat, carbohydrate metabolism and lipids. In addition, ALK7 and INHBB expression correlated but only in lean subjects and in subjects with normal glucose tolerance.

We conclude that activin B may have local effects in adipose tissue and thereby influence obesity and its comorbidities.

## Introduction

Activins were first described as gonadal hormones and their effects on reproduction have been extensively studied. Recent studies show that activins are also produced by extragonadal tissues where they have diverse effects [1]. Activins are formed by homo or heterodimerization of related beta subunits, resulting in several different activins [1]. In a search for adipocyte-specific genes we found that the inhibin beta B gene (INHBB) is highly expressed in human adipocytes and the corresponding protein is present in the adipocyte cytoplasm [2]. Intracellular homodimerization of two INHBB subunits results in formation of activin B, and the high expression of the INHBB gene in adipocytes therefore suggest that adipose tissue is a major site of activin B production.

Adipose tissue secretes a large variety of bioactive molecules, often referred to as adipokines. The adipokines have local effects or signal to other tissues, and they may play a central role in obesity-related morbidities such as diabetes, cardiovascular disease, cancer and dyslipidemia [3]. Genes that are specifically expressed in a tissue are likely to influence important functions, and genes that are highly expressed in adipocytes may, therefore, affect adipose tissue function or obesity related disorders. The unexpectedly high expression of INHBB in human adipocytes, therefore, raises the question of how and where the novel adipokine activin B exerts its action.

Activins belong to the TGF-β family of growth factors [1], and most members of this family act in a paracrine or autocrine fashion. The high expression of the INHBB subunit in adipose tissue may contribute to circulating levels of activin B. However, activins in the blood are bound to carrier proteins. Follistatin forms biologically inactive complexes with activin A and also reduces the activity of activin B [4,5]. Thus, it is likely that adipose tissue derived activin B exerts its effects locally and this requires the expression of signaling molecules.

Activins interact with receptor complexes consisting of two receptors, type I and II, both of which are serine/threonine kinases [6]. There are seven type I receptors, referred to as activin receptor-like kinases (ALK) 1 to 7, and ALK4 and ALK7 appear to function as type 1 receptors for activin B [1,6]. Activin receptor type II (ActR-II) and ActR-IIB are the main type II receptors for activins [1,6]. We have previously reported that some of the activin receptors are expressed in human adipose tissue [2] but more detailed analysis is lacking. The aim of this study was therefore to determine if human adipose tissue expresses receptors needed for paracrine or autocrine action of activin B.

## Methods

*Subjects.* The SOS Sib Pair study includes 154 nuclear families with BMI discordant sibling pairs (BMI difference ⩾10 kg/m^2^) resulting in a study population of 732 subjects. In this study, the most extreme siblings according to BMI were chosen in each family. Gender discordant sib pairs were excluded, resulting in 78 pairs of sisters and 12 pairs of brothers.

For tissue distribution, adipose tissue biopsies from six healthy volunteers (BMI range 22.4–29.3) were obtained. Adipocytes were isolated as previously described [7,8]. Subjects received written and oral information before giving written informed consent. The Regional Ethics Committee in Gothenburg approved the studies. Samplings and examinations were performed after an over night fast.

*Tissue distribution of gene expression.* DNA microarray expression profiles (Human Genome U133 plus 2.0, Affymetrix, Santa Clara, CA) from 65 human tissues were acquired from the GEO database (Dataset GSE3526; http://www.ncbi.nlm.nih.gov/geo/). Each tissue was represented by profiles from 3 to 9 subjects and these were used to calculate an average expression profile. For inhibin genes and activin receptor genes, the average expression in the 65 tissues was calculated and used for comparison with the adipose tissue expression. Probe sets were identified using Nettaffx (http://www.affymetrix.com/analysis/index.affx) and for each gene, the probe set with the highest signal was used. The following probe sets were used; INHA (210141_s_at), INHBA (210511_s_at), INHBB (205258_at), INHBC (207687_at), INHBE (210587_at), ALK7 (1552519_at), ALK4 (205209_at), ActRII (205327_s_at) and ActRIIB (236126_at).

For verification of tissue distribution, RNA from adipose tissue and adipocytes (prepared with RNeasy Lipid Tissue Mini Kit; Qiagen, Chatsworth, CA) and from the Human Total RNA Master Panel II (Clontech Laboratories, Inc., Palo Alto, CA), was reversed transcribed using the High Capacity cDNA RT kit (Applied Biosystems, Foster City, CA). Reagents for real-time PCR analysis of ALK7 (Hs00377065_m1) and peptidyl-prolyl isomerase A (PPIA; endogenous control, 4326316E) were from Applied Biosystems. cDNA corresponding to 10 ng RNA per reaction was used for real-time PCR in the ABI PRISM 7900HT Sequence Detection System (Applied Biosystems). Serial dilution of cDNA synthesized from pooled RNA was used to generate standard curves. PPIA expression was used to normalize ALK7 expression between samples. All samples were analyzed in triplicate.

*Microarray analysis in the SOS Sib Pair study.* Adipose tissue was obtained by needle aspirations in the paraumbilical area. Total RNA, cDNA and hybridization (Human Genome U133 plus 2.0, Affymetrix) was performed as previously described [7–9]. Data were analyzed using RMA. ALK7 expression was analyzed using probe set 1552519_at and INHBB was analyzed using 205258_at.

*Measurements in the SOS Sib Pair study.* Measurements of anthropometry, fat mass (FM), fat-free mass (FFM), blood pressure (BP), fasting glucose, total cholesterol, triglycerides, high-density lipoprotein cholesterol (HDL-C), low-density lipoprotein cholesterol (LDL-C), serum insulin, serum C peptide, and highly sensitive C-reactive protein (hs-CRP) were performed at the Sahlgrenska University Hospital. Dual-energy X-ray absorptiometry (DEXA) was performed with LUNAR DPX-L (Scanexport Medical, Helsingborg, Sweden). The DEXA generates a three-compartment model consisting of FM, lean tissue mass (LTM), and bone mineral content (BMC). The FFM was calculated as LTM + BMC.

*Statistics.* Statistical analyses were performed using SPSS (version 16.0; SPSS, Chicago, IL, USA) and SAS (version 9.1). Values are given as means ± SD unless stated otherwise. Correlation between ALK7, INHBB expression and anthropometric and biochemical markers were performed using the Spearman rank correlation test. Partial correlation was used to control for sex, age and fat mass when appropriate. In order to obtain approximate normal distributions of expression data, microarray signals in the whole SOS Sib Pair study offspring cohort (*n* = 359) were transformed using Box–Cox power transformations. Subsequently, expression data were standardized to mean = 0 and variance = 1. Differences in gene expression between lean and obese siblings were assessed using a paired *t*-test. A *P* value less than 0.05 (two-sided) was considered statistically significant. Linear relationships between ALK7 and INHBB transcript levels that differed between subgroups (lean vs. obese, or insulin resistant vs. insulin sensitive) were assessed in generalized linear models in which subgroup class was included as a covariate beside the transcript level. A *p*-value <0.05 for the interaction between subgroup class and transcript level was taken as evidence of a significantly different linear relationship between subgroup classes.

## Results

### Adipose tissue expression of activin subunits and activin receptors

**Fig. 1:**
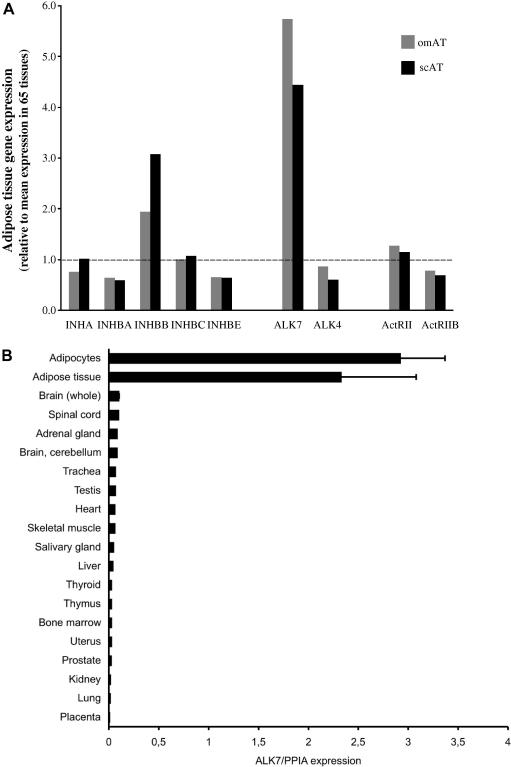
Expression of activin subunits and activin receptors in human adipose tissue analyzed by microarray (A) and ALK7 expression analyzed by real-time PCR (B). (A) For each gene, the expression in subcutaneous (scAT) and omental adipose tissue (omAT) relative to the mean expression level in 65 reference tissues (dashed line) is shown. (B) ALK7 gene expression in human adipose tissue (mean ± SEM, *n* = 3) and adipocytes (mean ± SEM, *n* = 3) compared with other human tissues.

Adipose tissue expression levels of inhibin genes and activin receptor genes were measured and compared with the mean expression levels of these genes in 65 different human tissues, hereafter referred to as reference tissues. In line with our previous results [2], expression of the INHBB gene was high in adipose tissue compared to the reference tissues (Fig. 1A), while the genes encoding the other activin subunits displayed adipose tissue expression levels equal to or lower than the mean expression in the reference tissues.

Among the type I activin receptor genes, ALK4 and ALK7 are known to mediate activin B action [6,10]. The adipose tissue expression of the ALK4 gene was lower than the mean expression in the reference tissues. In contrast, the ALK7 gene displayed the highest expression in human adipose tissue with more than 4-fold higher expression than in the reference tissues. ALK7 expression was also detected in mammary gland and in some regions of the brain, including putamen, nucleus accumbens and hippocampus (data not shown). The high expression of the ALK7 gene in human adipose tissue was verified by real-time PCR using a panel of 18 human tissues. In both human subcutaneous adipose tissue and isolated human adipocytes, ALK7 expression was markedly higher than in the other tissues (Fig. 1B).

Of the type II receptors, the adipose tissue expression of the ActRII gene was slightly above and the adipose tissue expression of the ActRIIB gene was slightly below the mean expression in the reference tissues.

### Adipose tissue expression of ALK7 and INHBB in lean and obese siblings in relation to clinical parameters

**Fig. 2:**
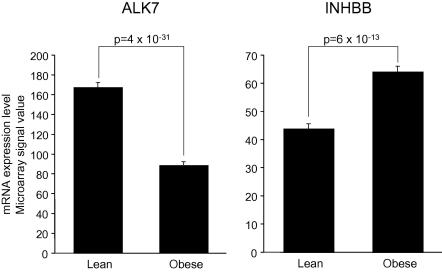
ALK7 (left) and INHBB (right) expression in lean (*n* = 90) and obese (*n* = 90) siblings from the SOS Sib Pair study measured by DNA microarray.

The high expression of genes encoding the activin receptor ALK7 and the activin subunit INHBB in human adipose tissue opens the possibility that locally produced activin B influences adipose tissue function. We therefore examined ALK7 and INHBB expression in relation to obesity and components of the metabolic syndrome. In the SOS Sib Pair study, adipose tissue ALK7 expression was markedly decreased in the obese compared to the lean siblings (*p* = 4 × 10^−31^). In contrast, INHBB expression levels were increased in the obese compared to the lean siblings (Fig. 2, *p* = 6 × 10^−13^).

**Fig. 3:**
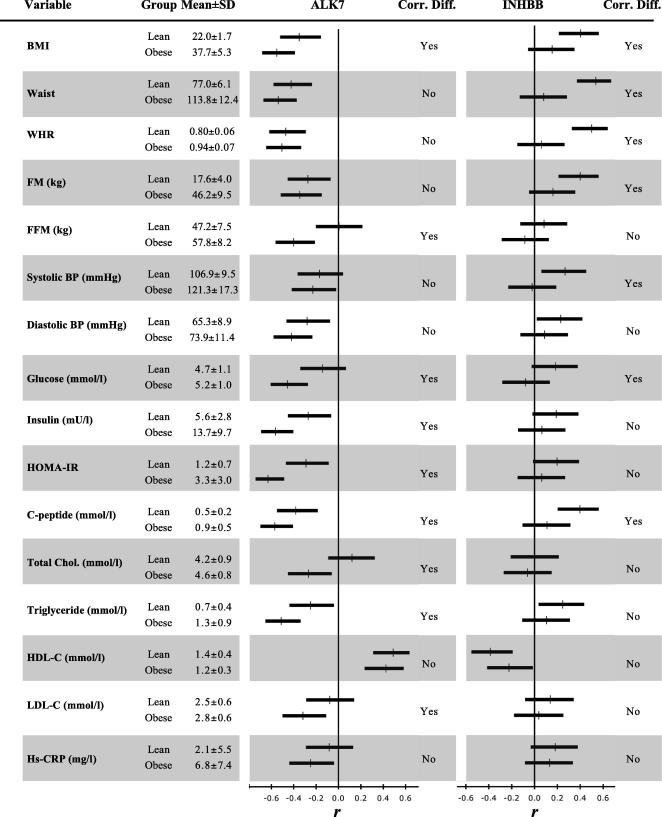
Spearman rank correlations between ALK7 and INHBB transcript levels and clinical traits in lean (*n* = 90) and obese (*n* = 90) subjects. Correlation coefficients (*r*) and their 95% confidence intervals, as derived from Fisher’s z-transformation, are represented by vertical marks and horizontal lines, respectively. Correlations are statistically significant if their confidence interval does not include zero (vertical axes). Corr. Diff. indicates whether correlations between clinical parameters and ALK7 or INHBB were significantly different between lean and obese subjects. Differences in *r* between lean and obese are significant if either confidence interval does not include *r* of the other.

Fig. 3 shows the correlations between ALK7 and INHBB expression and clinical parameters in the SOS Sib Pair study, as well as the difference between the correlation coefficients for each transcript in lean and obese subjects.

With the exception for a strong positive correlation with HDL-C, there were significant negative correlations between ALK7 and clinical parameters in both groups. For measures of glucose and lipid metabolism, the correlation coefficients for ALK7 were different in lean and obese subjects.

For INHBB, there were positive correlations with clinical parameters in the lean subjects, whereas the correlations were considerably weaker or lost in the obese subjects. In addition, there was a negative correlation between HDL-C and INHBB expression in both groups. The correlation coefficients for INHBB and measures of fat mass differed in lean and obese groups.

### Correlations between adipose tissue ALK7 and INHBB expression levels

**Fig. 4:**
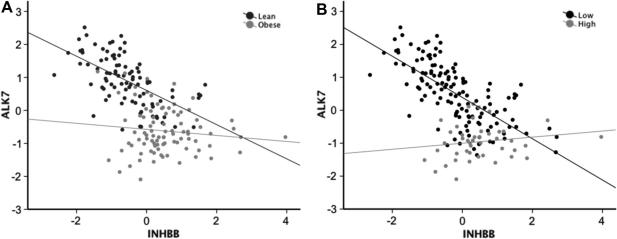
Correlations between INHBB and ALK7 gene expression. (A) The relation between INHBB and ALK 7 in lean (*n* = 90) and obese (*n* = 90) siblings. (B) The relation between INHBB and ALK7 in subjects in the highest quartile (*n* = 44) with respect to serum insulin levels compared to the rest of the group (*n* = 134). The slopes for the relation between INHBB and ALK7 expression were significantly different in lean and obese (*p* < 0.001) as well as between subjects in the highest quartile with respect to serum insulin levels compared to the rest of the group (*p* < 0.001). Data are standardized to mean = 0 and SD = 1.

When the relationship between ALK7 mRNA and INHBB mRNA was analyzed there was a negative correlation in the lean group (*r* = −0.64, *p* = 7 × 10^−12^), and this persisted after controlling for sex and age (*r* = −0.58, *p* = 2 × 10^−9^). In the obese subjects, there was no correlation (*r* = −0.03, *p* = 0.8; Fig. 4A) even after controlling for sex and age (*r* = −0.16, *p* = 0.15), and the slope was significantly different for the lean vs obese (*p* < 0.001; Fig. 4A) according to a generalized linear model. Thus, the relationship between the ligand, encoded by INHBB, and the receptor, encoded by ALK7, was different in lean and obese subjects. The correlations between INHBB and measures of body fat differed between lean and obese groups (Fig. 3). To determine if obesity influenced the relationship between the two transcripts, we performed the analysis after controlling for fat mass in addition to sex and age and found that there was still a significant correlation in the lean group (*r* = −0.52, *p* = 2 × 10^−7^) and no correlation in the obese group (*r* = −0.17, *p* = 0.13). There were also several differences in the relation between gene expression levels and measures of carbohydrate metabolism in the two groups (Fig. 3). We therefore subdivided all subjects into quartiles of the HOMA-IR index. In the subjects with the highest HOMA-IR index, there was no correlation between ALK7 expression and INHBB expression. In marked contrast, in the three other quartiles there were highly significant correlations between ALK7 and INHBB expression (*p* < 2 × 10^−5^ for each of the three quartiles after adjusting for sex and age). Since HOMA-IR is derived from glucose and insulin levels, we next performed the same calculations for insulin and glucose quartiles. For insulin, the pattern was strikingly similar to that seen for HOMA-IR, i.e. a loss of correlation between ALK7 and INHBB expression in the highest quartile (*r* = 0.12, *p* = 0.4) and significant correlations in the remaining quartiles (*r* = −0.63, *p* = 5 × 10^−6^; *r* = −0.64, *p* = 6 × 10^−6^; *r* = −0.61, *p* = 1 × 10^−5^, respectively). Hence, the slope was significantly different between the subjects from the three lowest insulin quartiles compared to the subjects from the highest insulin quartile (*p* < 0.001; Fig. 4B), according to a generalized linear model. For glucose, there were significant correlations between the two genes (*p* ⩽ 0.001 for each quartile after adjusting for sex and age).

## Discussion

In this study, we have extended our previous observation that the beta B subunit, which homodimerizes to form activin B, is predominantly expressed in human adipose tissue [2] and here demonstrate that the activin B receptor ALK7 is adipocyte specific. There were highly significant differences in the expression of both ALK7 and INHBB in lean and obese subjects and ALK7 and INHBB expression correlated with several indicators of the metabolic syndrome. In addition, the expression of the two transcripts correlated but only in lean subjects and subjects with normal glucose tolerance.

Activins are involved in various physiological processes, such as cell proliferation, immune function, wound repair and reproduction, and exert their effects in many tissues [1,4,6]. Our data clearly show that receptors required for activin B signaling are expressed in adipose tissue. The findings that both ALK7 and INHBB are expressed at high levels in adipocytes and that the adipocyte is their main site of expression indicate that the activin B signaling system is important for adipose tissue function. This idea is supported by our observation that both INHBB and ALK7 expression are highly regulated by obesity and the expression levels correlate with metabolic parameters. Furthermore, our study shows that there is an altered relationship between ALK7 and INHBB expression in subjects with hyperinsulinemia, opening the possibility that activin B signaling is involved in metabolic control or that insulin affects the expression.

ALK7 expression has previously been detected in the brain, pancreas and colon, however, adipose tissue was not included in the analysis [11]. In rodents, ALK7 expression is expressed in several organs including brain [12], ovary [13], pancreas [14] and adipose tissue [15]. In our study, both microarray analysis and real-time PCR showed that adipose tissue and adipocytes display the highest expression of ALK7. In the analysis of microarray profiles from a large number of human tissues we also found high expression of the ALK7 gene in the mammary gland. However, in mammary gland microarray profiles, high expression of adipocyte specific markers, such as leptin and adiponectin, was also observed, strongly suggesting that the high ALK7 gene expression was due to the presence of adipose tissue in the biopsies. The very high and specific expression of ALK7 in adipose tissue suggests that signaling through this receptor influences adipose tissue function. In a very recent report, it was shown that disease genes often are specifically expressed in the normal tissue corresponding to the tissue affected by the disease [16], supporting the idea that ALK7 could be involved in obesity and metabolic disease.

Recent studies in rodents and *in vitro* also support our hypothesis that the activin B receptor ALK7 is implicated in the regulation of metabolism and adipose tissue function. ALK7 is specifically expressed during the late phase of adipocyte differentiation [17], suggesting that activin B is involved in adipogenesis. ALK7 deficient mice have hyperinsulinemia, reduced insulin sensitivity and impaired glucose tolerance [15,18]. Interestingly, in our study there were negative correlations between ALK7 expression serum insulin, HOMA, C-peptide and plasma glucose which is in line with the observations in the ALK7 deficient mice. However, mice lacking ALK7 are partially resistant to diet-induced obesity, have smaller adipocytes, smaller epididymal fat pads and increased accumulation of lipids in the liver. In human subjects, we found negative correlations between ALK7 expression and obesity, indicating that there may be species differences or that a complete loss of function is required to affect the amount of adipose tissue and adipocyte size. In our study, ALK7 expression correlated with serum lipid levels but no data are available on serum lipids in AKL7 deficient mice.

In conclusion, human adipose tissue expresses receptors required for paracrine or autocrine effects of activin B. The effects of obesity on adipose tissue expression of ALK7 and INHBB genes, the strong correlations with markers of the metabolic syndrome and the dysregulation between ligand and receptor transcripts in subjects with glucose intolerance indicate that the adipokine activin B has hitherto unrecognized metabolic effects.
